# Practical Application of Evidence-Based Dietary Therapy in Inflammatory Bowel Disease: The DELECTABLE Program

**DOI:** 10.3390/nu17091592

**Published:** 2025-05-06

**Authors:** Gina L. Trakman, Erin E. Russell, Amy L. Hamilton, Amy Wilson-O’Brien, Emily Thompson, Natalie Simmance, Ola Niewiadomski, Michael A. Kamm

**Affiliations:** 1 Department of Gastroenterology, St Vincent’s Hospital, Melbourne 3065, Australia; g.trakman@latrobe.eud.au (G.L.T.); erin.russell@svha.org.au (E.E.R.); amy.hamilton@svha.org.au (A.L.H.); ola.niewiadomski@svha.org.au (O.N.); 2 Department of Medicine, The University of Melbourne, Melbourne 3065, Australia; 3 Discipline of Food, Nutrition and Dietetics, Department of Sport, Exercise and Nutrition Science, La Trobe University, Melbourne 3086, Australia; 4 Department of Nutrition and Dietetics, St Vincent’s Hospital, Melbourne 3065, Australia; emily.thompson@svha.org.au (E.T.); natalie.simmance@svha.org.au (N.S.)

**Keywords:** compliance, tolerance, emulsifier, Mediterranean diet, inflammation

## Abstract

Background/Objectives: Exclusive Enteral Nutrition (EEN) and the Crohn’s Disease Exclusion Diet (CDED) have been shown to induce remission in Crohn’s disease. Low-sulphur, plant-based diets are being explored for ulcerative colitis, and wholefood, low-additive approaches are emerging as significant. Although Inflammatory Bowel Disease (IBD) patients modify their diet, evidence for tolerability and benefit outside clinical trials is limited. The DELECTABLE program aimed to assess satisfaction, adherence, and efficacy of dietary therapies as part of IBD care. Methods: In this dietitian-led, open-label, prospective study, patients with Crohn’s disease were offered the CDED or a whole-food, additive-free diet (WFD), and patients with ulcerative colitis were offered a low-sulphur, plant-based diet (UCD) or WFD. Primary outcomes were 12-week diet satisfaction (modified DSAT-28) and diet adherence, including food additive intake. Secondary outcomes were quality of life (QoL) (IBDQ-9), disease activity (CDAI for Crohn’s disease, partial Mayo score for ulcerative colitis), and biochemical markers (CRP, faecal calprotectin). Analyses were conducted within, rather than between, diet arms due to the non-random nature of the study. Diet adherence and disease activity change across time points (baseline, week 6, week 12) were assessed using repeated measures ANOVA or Friedman’s test, with pairwise paired *t*-test or Wilcoxon Signed-Rank test. Diet satisfaction and quality of life changes across time (baseline/week 1, week 12) were assessed using a paired *t*-test or Wilcoxon Signed-Rank test. Results: Of 165 referrals, 76 patients enrolled, with 64 completing the 12-week program (CDED: *n* = 15, WFD: *n* = 42, UCD: *n* = 7). Diet satisfaction was initially high and remained stable over time on CDED (*p* = 0.212) and improved on WFD (*p* = 0.03). Patient- and dietitian-rated adherence was high at baseline and did not significantly decrease on any diet arm (*p* > 0.349). Food additive intake decreased on WFD (*p* = 0.009). QoL improved on CDED and WFD (*p* < 0.001). CRP, calprotectin, and CDAI were reduced on CDED (*p* < 0.045), and CDAI and partial Mayo were reduced on WFD (*p* < 0.027). Conclusions: Well-balanced therapeutic diets are feasible and well-accepted by patients with IBD, with a promising impact on disease activity.

## 1. Introduction

Inflammatory Bowel Diseases (IBD), predominantly Crohn’s disease and ulcerative colitis, are immunologically mediated, chronic, relapsing conditions resulting from interactions between the gut microbiota and the environment [1]. Immune-suppressing drugs, the current mainstay IBD therapy, are not always effective, can result in adverse side effects [2], and are associated with variable adherence [3].

Exclusive Enteral Nutrition (EEN), a specialised liquid nutrition formula diet, can induce remission and heal the mucosa in paediatric [4] and adult [5] Crohn’s disease. EEN is recognized by the American Gastroenterology Association (AGA) as an effective approach for the induction of remission in Crohn’s disease [6]. However, it is limited by low tolerability and adherence, especially when not administered by a dietitian [7,8]. Furthermore, EEN is not suitable long-term [9]. However, patients have a keen interest in diet [10,11] and often self-restrict their intake to control symptoms and in an attempt to diminish disease activity [11,12,13].

Limitations of EEN, patients’ interest in diet, and literature demonstrating specific food elements that may play a role in the pathophysiology of active disease [14,15,16,17,18,19,20] have led to an interest in food-based dietary therapies [15,21], such as the Crohn’s Disease Exclusion Diet (CDED). The CDED excludes the intake of foods believed to be associated with inflammation, microbial dysbiosis, and gut symptoms (for example, food additives [22,23], red meat, dairy, and wheat products [15,24]) and promotes mandatory daily intake of foods that support the growth of beneficial gut bacteria (for example, bananas, potatoes, and apples [25]). In a randomised controlled trial (RCT), the CDED with Partial Enteral Nutrition (PEN) over 12 weeks was equally effective but better tolerated than EEN alone in adolescents with mild to moderate luminal Crohn’s disease [25], with follow-up studies indicating CDED plus PEN induces remission in approximately 70% of adults [26,27]. The CDED has been adopted into clinical practice, but concerns remain regarding the diet’s restrictive nature, lack of evidence in severe disease, and the duration of benefit [28].

Wholefood diet approaches are appealing as they are less restrictive than EEN or the CDED, and therefore, potentially more sustainable long-term and likely to be better tolerated [29]. Wholefood approaches, such as the Mediterranean diet, typically include a wide variety of nutrient-dense unprocessed or minimally processed plant foods, dairy, and healthy fats, and leads to incidental reduction of food additive-containing ultra-processed foods. The AGA recommends that, unless contradicted, all patients with IBD follow a Mediterranean diet [6]. Likewise, wholefood approaches align with dietary guidance from the International Organization for the Study of Inflammatory Bowel Diseases (IOBD), which recommends that it is prudent to reduce intake of trans fats, red and processed meat, and processed foods containing artificial sweeteners, emulsifiers (carboxymethylcellulose, polysorbate 80), carrageenan, and nanoparticles (titanium dioxide, sulphites) [24].

Food additive restriction has been proposed to be effective for inducing and maintaining remission in IBD [21,30] because of the association between additive intake and IBD incidence [31,32], studies demonstrating increased ad libitum intake of food additives in individuals with IBD compared to controls [32,33], and evidence from pre-clinical studies that food additives are associated with immune activation, inflammation, and impaired gut-barrier function [16,17,18,19,20]. 

A RCT comparing the Specific Carbohydrate Diet, a restrictive diet similar to the CDED, to a more liberal Mediterranean diet [29], found comparable symptom and inflammation reduction in patients with mild to moderate Crohn’s disease. The study concluded that the Mediterranean diet is preferred as the least restrictive dietary option but is limited by the lack of a control diet to show that either diet is superior to a standard diet in Crohn’s disease [29]. In relation, a pilot RCT comparing an anti-inflammatory, low-additive diet, with similarities to the Mediterranean diet, to general healthy-eating education in patients with IBD found equivalent week 8 CRP reductions in ulcerative colitis and Crohn’s disease patients and equivalent disease score reductions in Crohn’s disease patients [34].

Evidence for dietary therapy in inducing remission in ulcerative colitis, microscopic colitis [35], and pouchitis [36] is less robust, with low-sulphur and plant-based diets currently being investigated in ulcerative colitis [37]. These approaches are supported by pre-clinical evidence [38,39,40,41] but are not used in routine clinical practice.

Although the rationale for choosing food-based approaches over well-established EEN relates to the low tolerability of liquid nutrition, there is limited evidence on patients’ diet satisfaction, adherence, and quality of life on these approaches outside of clinical trials. Likewise, the impact of dietary approaches for patients who are failing medication and have more severe Crohn’s disease, colonic Crohn’s disease, ulcerative colitis, microscopic colitis, pouchitis, or quiescent disease is unknown. Access to structured, evidence-based dietary support outside of clinical trials is lacking.

To address some of these gaps, we aimed to implement and evaluate a structured, ongoing, dietitian-led telehealth service for patients with IBD who had a spectrum of disease activity. We hypothesized that these diets would be satisfying, well-tolerated, improve quality of life, and reduce disease activity for patients who enrolled in the program.

## 2. Materials and Methods

### 2.1. Study Design and Setting

This was an open-label, prospective, longitudinal, interventional cohort program in which three diets were offered to patients with IBD (Crohn’s disease, ulcerative colitis, chronic pouchitis, and microscopic colitis). The diet arms were the Crohn’s Disease Exclusion Diet (CDED) with partial enteral nutrition (PEN) (hereafter, CDED), the “Wholefood Diet” (WFD) and the “Ulcerative Colitis Diet” (UCD). The latter 2 are novel diets designed by the study team and based on currentliterature (see Section 2.2).

**Figure 1:**
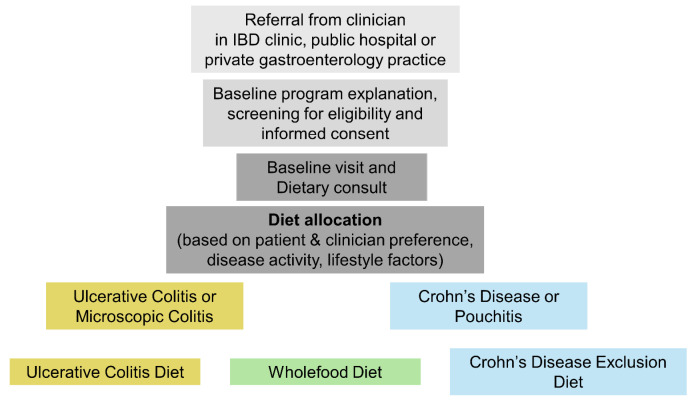
Process for referral and diet allocation in the DELECTABLE program.

The diet choice for each patient was based on shared decision-making between the patient, dietitian, and managing clinician at the baseline visit. The key factors influencing diet choice were disease diagnosis and phenotype, disease activity, habitual diet before program entry, lifestyle, and patient preference (Supplementary Table S1). Crohn’s disease and pouchitis patients were allocated to the CDED or WFD, and patients with ulcerative colitis were allocated to the WFD or UCD (Figure 1). The CDED and WFD were options for pouchitis patients based on available safety and efficacy data [42]. Given the lack of evidence for dietary therapy in microscopic colitis, these patients were allocated to the WFD, which aligns with national dietary guidelines.

The intervention was initially intended to be a hybrid of in-person and telehealth. However, due to the COVID-19 pandemic, all visits were conducted via telehealth.

Referrals came from treating gastroenterologists at tertiary hospital IBD clinics and private practices, and patients were recruited between November 2021 and February 2023 in Victoria, Australia.

**Figure 2:**
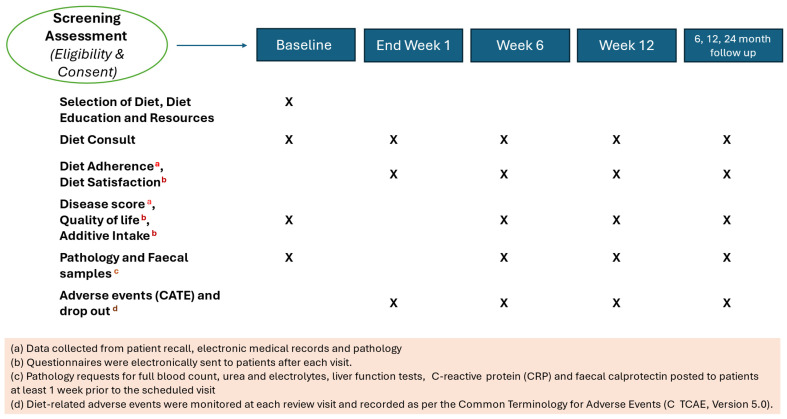
Study visits and outcome measure timetable for The DELECTABLE Program.

At baseline, weeks 1, 6, and 12 visits, patients attended appointments with a dietitian, and additional reviews were offered as needed. Participants were then followed prospectively for 12 months, with additional long-term follow-up scheduled at 24 months (Figure 2). Here, we report data for weeks 6 and 12 for all outcomes, as well as 12-month data for the primary outcome measures (patient-rated compliance, dietitian-rated compliance, and diet satisfaction).

### 2.2. Dietary Interventions

#### 2.2.1. Crohn’s Disease Exclusion Diet

The Crohn’s Disease Exclusion Diet (CDED) is a 3-phase diet that has been shown to induce remission and reduce inflammation in mild to moderate Crohn’s disease. The diet and rationale for inclusion and exclusion of specific foods have been described in detail elsewhere. In brief, the diet consists of two six-week phases, during which there are daily mandatory foods, a short list of additional foods, and 25–50% of energy intake is from oral nutrition supplements [25]. Data beyond 12 weeks on the diet are limited [28]. In one study that followed patients to 24 weeks, after Phase 2 of the CDED, patients were advised to follow the CDED guidelines for five days of the week and consume some disallowed foods in moderation on the other two days [26]. In contrast, expert practical guidance on using the CDED in Australian adults [28] recommends that after Phase 2 of the CDED, patients transition to a well-balanced diet that follows the Australian Guide to Healthy Eating [28] or (ii) transition [43]. No data are available to compare these approaches. In the current DELECTABLE program, all patients were advised to transition to the WFD at Week 12.

#### 2.2.2. Wholefood Diet

Designed by the DELECTABLE research group, the WFD is based on two key principles that aim to optimise the gut microbiota.

The first principle is the exclusion of food additives, some of which have been shown to have deleterious effects on the gut microbiota [16,17]. For example, there is evidence that exposure to emulsifiers [16,17], thickeners [44], and artificial sweeteners [45,46] changes the abundance and pathogenicity of anti- and pro-inflammatory gut bacteria in humans, animals, and ex-vivo, and perturbs human and rodent gut mucosa. These additives have also been hypothesised to increase the risk of developing several chronic diseases, including IBD [47,48].

The second principle is the inclusion of a wide variety of nutrient-dense wholefoods, including fruits, vegetables, whole grains, nuts and seeds, unprocessed dairy, and olive oil. Fruits, vegetables, and whole grains are high in fibre, micronutrients, and antioxidants; these food components have been shown to increase the abundance of beneficial, butyrate-producing bacteria that are typically depleted in IBD, such as *Bifidobacterium*, *Roseburia*, and *Dialister* [49]. Likewise, polyphenol-rich olive oil, fruits, and vegetables have been shown to increase the abundance of *Akkermansia muciniphila*. This bacterium is often reduced in inflammatory bowel disease (IBD) and is negatively correlated with various metabolic disorders [50].

Regarding protein intake, the WFD recommends chicken, eggs, and plant-based proteins (nuts, legumes) as primary sources and allows for the inclusion of unprocessed dairy, a direct source of *Bifidobacterium*. Lean chicken and eggs are nutrient-dense and contribute to overall nutritional adequacy. While there is limited research on their specific impacts on the microbiome, they are lower in saturated fat than other animal protein sources. Therefore, their inclusion in the diet may mitigate some of the adverse effects associated with high saturated fat intake, such as the expansion of pro-inflammatory, bile-tolerant bacteria like *Bacteroides* and *Allistipes* [38,41]. Nuts and beans, being rich in fibre and antioxidants, have been associated with increased levels of anti-inflammatory, butyrate-producing bacteria, including *Clostridium*, *Dialister*, *Lachnospira*, and *Roseburia* [49].

#### 2.2.3. Ulcerative Colitis Diet

The UC diet was commensurate with the above WFD, with an additional focus on reducing intake of fat and moderating intake of sulphite additives, sulphur-containing vegetables, and sulphur-containing amino acids (cysteine, taurine, methionine) that are typically found in animal products. Patients were advised to limit the intake of red meat, eggs, fish, saturated fat, and certain cruciferous vegetables and encouraged to have some ‘meat-free’ days, although daily intake of chicken breast was permitted.

The rationale for these guidelines relates to dietary saturated fat and microbial sulphur metabolism. Saturated fat promotes the expansion of sulphate-reducing bacteria, and animal proteins are a key source of dietary sulphur. Sulphate-reducing bacteria use sulphur and sulphate for energy and produce harmful metabolites such as hydrogen sulphide, which are postulated to break di-sulphide bonds in the gut mucus layer and impair intestinal barrier integrity [40,51]. This disruption in the intestinal barrier function is believed to be the hallmark of UC pathophysiology, through increased microbial adherence and antigen capture [39].

Nutritional adequacy was assessed for all diets by comparing sample meal plans of each diet to (i) the Australian Guide to Healthy Eating [minimum Core Food Group Requirements], (ii) Nutrient Reference Values (NRVs) for macronutrients [Acceptable Macronutrient Distribution Range], and (iii) NRVs for micronutrients [Recommended Dietary Intake, Adequate Intake, Upper Level] [52] for both males and females of healthy BMI and activity level (Supplementary Table S2).

### 2.3. Inclusion and Exclusion Criteria

Patients were eligible to participate if they were ≥18 years of age and had active or quiescent IBD. Patients were able to commence new drug therapies while on the program. Exclusion criteria were a diagnosis of a Diagnostic and Statistical Manual (DSM)-defined eating disorder or dietitian concerns about restrictive eating behaviour, acute bowel obstruction requiring surgical intervention, and suspected perforation of the gastrointestinal tract.

### 2.4. Outcome Measures

Outcome data were recorded and managed with REDCap [53,54]. Diet-related adverse events were monitored at each review visit, with details of events recorded as per the Common Terminology for Adverse Events (CTCAE, Version 5.0). Questionnaires were electronically sent to patients after each visit. All other data were collected from patient recall during telehealth appointments, referral letters, electronic medical records, and pathology reports. Figure 2 provides an overview of outcomes measured at each study assessment.

The primary endpoints were week 12 adherence and diet satisfaction. Secondary endpoints included clinical efficacy (biochemical markers of disease activity and clinical disease activity scores) and quality of life.

#### 2.4.1. Adherence

Patient-rated adherence was assessed using a modified Medication Adherence Report Scale and CDED adherence questionnaire [3,55]. In the latter, patients rated adherence on a 6-point Likert scale (Never to Always).

Dietitians’ assessment of adherence was based on direct questioning and 24-h diet recall, rated on a 5-point Likert scale (non-adherent to very adherent) (Supplementary Table S3).

Given that all diets were designed to reduce food additive intake, food additive intake, measured using a validated food-additive frequency questionnaire (FFQ), was included as an additional measure of adherence. The FFQ was developed and published by the research team [56]. The questionnaire addresses intake frequency and amount over the past 12 months of 23 processed food groups. Maximal exposure to food additives is then estimated in mg/year, based on the maximal permissible level of additives in the food, or concentration data from the literature. The published FFQ has undergone recent modifications to maintain contemporary relevance (Supplementary Table S4).

#### 2.4.2. Diet Satisfaction

Diet satisfaction was measured using a modified version of a validated diet satisfaction questionnaire (DSAT-28) (range of 27–135; higher scores indicate greater satisfaction) [57]. The original DSAT-28 was developed for use in weight loss diets and includes subscales to assess participant perception of food cost, preoccupation with food, planning and preparing meals, eating outside the home, and healthy lifestyle. Before implementing the DSAT-28 in this program, it was pilot-tested on five expert IBD dietitians, leading to the removal of four irrelevant items and the addition of three IBD-specific items (Supplementary Table S5).

#### 2.4.3. Clinical Efficacy

Clinical efficacy was assessed at baseline, week 6, and week 12 using biochemical markers of disease activity (CRP and faecal calprotectin), and clinical disease activity scores (Crohn’s disease activity index (CDAI) [58] for Crohn’s disease patients and partial Mayo score [59] for ulcerative colitis patients).

#### 2.4.4. Quality of Life

Quality of life was assessed using the validated IBDQ-9 (score range 7 to 63; with higher scores indicating better self-reported quality of life) [60].

### 2.5. Statistical Analysis

Continuous variables were reported as mean ± SD or median [IQR], and categorical variables were reported as counts and percentages. ANOVA or Kruskal–Wallis and chi-squared tests were applied to assess differences in continuous and categorical baseline demographics, respectively.

All other analyses were conducted within, rather than between, diet arms due to the non-random nature of the study. Diet adherence and disease activity change across time points (baseline, week 6, week 12) were assessed using repeated measures ANOVA or Friedman’s test, with pairwise paired t-test or Wilcoxon Signed-Rank test. Diet satisfaction and quality of life changes across time (baseline/week 1, week 12) were assessed using a paired t-test or Wilcoxon Signed-Rank test. Since only 7 participants were allocated to the UC diet, inferential analyses were not conducted.

Where data were missing, participants were excluded from paired analyses. Where participants were lost to follow-up, they were excluded from analyses.

A qualitative description of changes in disease activity was undertaken for participants who started new medication or had a dose escalation, and exploratory subgroup analyses of changes in disease scores and inflammatory markers were assessed for patients with active disease at baseline.

## 3. Results

### 3.1. Patient Enrollment and Allocation

**Figure 3:**
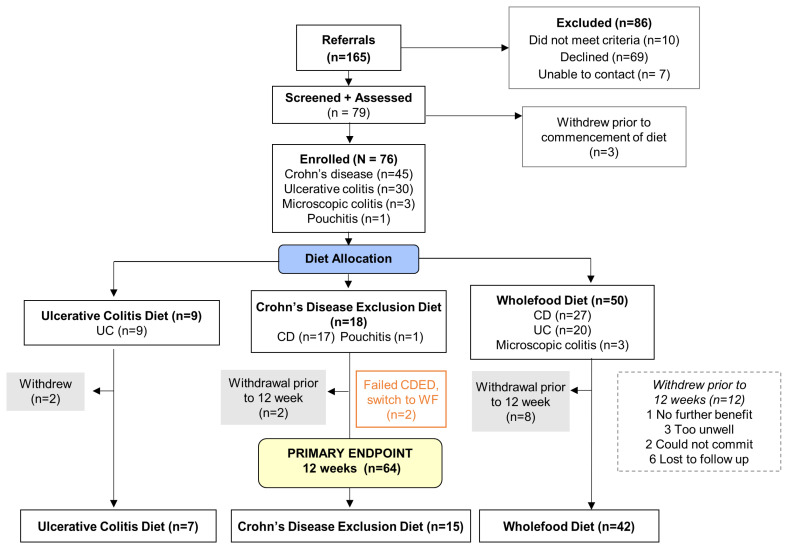
Consort diagram for the DELECTABLE program.

Of one hundred and sixty-five referred patients, eighty-six (52%) were excluded or declined, and three (2%) dropped out before commencing an intervention. The main patient-driven reasons for not enrolling included an inability to commit to regular and long-term follow-up, as well as a pre-existing diet that already approximated the recommended interventions. Of the 86 patients who did not enroll, some were seeking once-off dietary advice or could not commit to research activities long term. Seventy-six (46%) patients were allocated to a diet arm at Week 1, and sixty-four (84%) completed 12 weeks of dietary therapy (Figure 3). Data for 12-month adherence and satisfaction were available for 29 (45%) and 25 (40%) enrolled participants.

### 3.2. Demographics and Baseline Characteristics

Thirty-four of the seventy-six patients (53%) were male, with a median age of 36 years. Of the seventy-six enrolled participants, thirty-seven (58%) had Crohn’s disease, twenty-five (39%) had ulcerative colitis, and two (3%) had microscopic colitis. The WFD was the most common diet allocation (*n* = 42, 66%), followed by the CDED (*n* = 15, 24%) and the UCD (*n* = 7, 11%). No concomitant medications were being used in 3/15 (22%) and 14/42 (33%) of WFD and CDED patients, respectively. As expected, there was a significant difference in diet chosen across disease states (*p* < 0.001) (Table 1).

### 3.3. Short-Term (Week 1 to 12) Diet Adherence

Median dietitian-rated adherence scores were high at week 1 and did not change at week 12 on the CDED (*p* = 0.565), WFD (*p* = 0.165), and UCD (*p* = 0.233). Likewise, median patient-rated adherence scores were high at week 1 and did not change at week 12 on the CDED (*p* = 0.682), WFD (*p* = 0.348), or UCD (Supplementary Table S6).

At week 12, dietitians rated 85% (*n* = 13) of patients on the CDED, 80% (*n* = 34) on the WFD, and 100% (*n* = 7) on the UCD as “fairly adherent” or “very adherent”. At week 12, 85% (*n* = 13) of CDED, 67% (*n* = 28) of WFD, and 100% (*n* = 7) of UCD patients reported that they adhered to their diet “very often” or “always” (Supplementary Table S6). Total daily food additive intake (mg/day) was reduced on the CDED, but the result was not statistically significant (*p* = 0.441). The daily food additive intake was halved on the WFD (*p* = 0.009) and reduced by more than fourfold on the UCD (*p* = 0.018) (Table 2). On the WFD, there was a significant reduction in carboxymethylcellulose (*p* = 0.02), carrageenan (*p* = 0.013), sucralose (*p* = 0.018), and sulphites (*p* = 0.007).

### 3.4. Short-Term (Week 1 to 12) Diet Satisfaction

**Figure 4:**
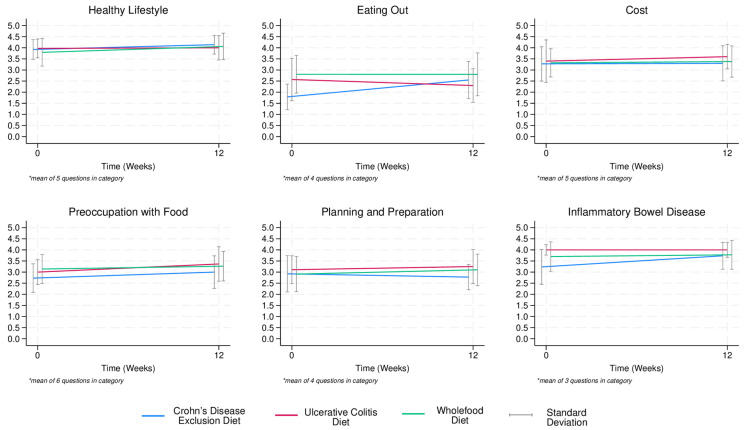
Line chart depicting diet satisfaction at week 1 and week 12 among patients allocated to the Crohn’s Disease Exclusion Diet (*n* = 15), the Ulcerative Colitis Diet (*n* = 7), and the Wholefood Diet (*n* = 42) on the DELECTABLE program. * Numbers represent mean of questions in item category.

Diet satisfaction was high at week 1 and did not significantly change between week 1 and week 12 on the CDED (*p* = 0.212). On the WFD, diet satisfaction improved from week 1 to week 12 (*p* = 0.03) (Supplementary Table S6). Within the DSAT-28 sub-scales, Eating Out scores improved on the CDED (*p =* 0.005), and Healthy Lifestyle (*p =* 0.050) and Planning and Preparation (*p =* 0.008) scores improved on the WFD (Supplementary Table S7, Figure 4).

### 3.5. Short-Term (Week 1 to 12) Clinical Efficacy

Disease activity indices improved during the baseline to week 12 study period as measured by the CDAI on the CDED (*p* = 0.023), with pairwise analyses revealing a decrease from baseline to week 6 (*p* = 0.014) and baseline to week 12 (*p* = 0.024). CDAI also decreased in Crohn’s patients on the WFD (*p* = 0.038), with pairwise analyses revealing a decrease from baseline to week 6 (*p* = 0.042) and baseline to week 12 (*p* = 0.027). There was a numerical decrease in the partial Mayo score, but the sample size was too small to assess significance (Table 3).

There was also a significant decrease in CRP across all baseline to week 12 timepoints (*p* = 0.043) on the CDED, with pairwise comparisons showing a significant decrease from baseline to week 12 (*p* = 0.034). There was a numerical decrease in CRP on the WFD and UCD, and in faecal calprotectin in each diet arm, but these reductions were not significant (Table 3).

**Figure 5:**
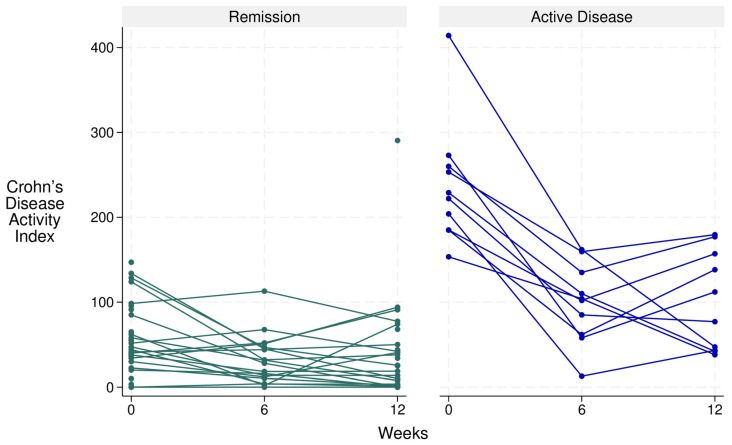
Line chart depicting disease activity of individual patients with Crohn’s disease at baseline, week 6, and week 12 on the DELECTABLE program.

In order to account for heterogeneity in disease activity at enrolment, we conducted a stratified analyses of participants in remission versus those with active disease at baseline. Patients with active disease at baseline exhibited a similar response pattern to those with inactive disease at baseline (Figure 5; Supplementary Table S8).

Given our program aimed to mimic routine clinical practice, we allowed for medication changes during the study period, but kept a record of these. A total of twenty-six patients underwent medication changes from Weeks 1 to 12, with nine commencing new medications, three reducing their medication dose, and fifteen ceasing medications (Supplementary Table S9). Most patients commencing biologic drugs were on the CDED, and disease activity improvements occurred before biologics began.

### 3.6. Short-Term (Week 1 to 12) Quality of Life

There was a significant improvement in quality of life (IBDQ-9) from baseline to week 12 on the CDED (*p* < 0.001) and the WFD (*p* < 0.001). Numerically, quality of life measures were stable or increased on the UCD; however, we did not apply any inferential statistics within this group due to the sample size (*n* = 7) (Supplementary Table S6).

### 3.7. Long-Term (Week 1 to 12 Months) Diet Adherence and Satisfaction

Neither 12-month dietitian-rated (*p* = 0.131) nor patient-rated (*p =* 0.429) adherence scores changed for patients who were initially allocated to the CDED and transitioned to the WFD at week 12. Similarly, there was no change in dietitian-rated (*p* = 0.882) or patient-rated adherence scores (*p =* 0.537) on the WFD, and no changes on the UCD (Supplementary Table S7).

There were no significant changes in total diet satisfaction on the CDED (*p* = 0.800), WFD (*p* = 0.759), and UCD (Supplementary Table S6).

## 4. Discussion

The DELECTABLE program implemented structured, practical dietary strategies in routine, real-world clinical care for all patients with IBD. The primary aim was to assess whether patients could adhere to, and were satisfied with, each approach. A secondary aim was to assess efficacy and evaluate quality of life.

### 4.1. Feasibility and Adherence

Half of the referred patients were eligible and interested in pursuing long-term dietary support; of these, 86% (*n* = 64) completed 12 weeks of diet therapy. Comparatively, in a feasibility study of preoperative CDED, 35% of screened patients were enrolled, of which 76% completed the 6-week intervention [61]. Our relatively high enrolment and completion rates indicate that the implementation of diet strategies into clinical practice is feasible.

Non-adherence is a major limiting factor for diet therapies. In the present program, patients who enrolled could adhere to strict (CDED, UC) and moderate (WFD) food-based approaches. Dietitian- and patient-rated adherence scores remained high for 12 weeks. They were maintained at 12 months in a subset of participants for whom data were available, though selection bias may have impacted the latter. Findings that 67% (*n* = 28) and 80% (*n* = 12) of patients followed the WFD and CDED ‘very often’ or ‘always’ at week 12 are aligned with self-reported data indicating 68% and 64% of participants followed the Mediterranean and Specific Carbohydrate diet “all of the time” [29], but lower than 88% reported adherence rates to the “tasty and healthy” diet [62], and 92% reported adherence rates to an anti-inflammatory, low additive diet [34]. Adherence rates for the WFD were higher than EEN, which has 33–41% reported non-adherence rates [5]. The relatively high adherence rates and reduction in food additive intake in the UCD and WFD arms in our study were likely improved by intensive dietitian support, as has previously been shown with EEN [5,7] and low-emulsifier diets [21], highlighting the importance of a strong dietitian presence in an IBD service.

Patients with IBD consume several additive-containing foods, including those with emulsifiers [32,33,63]. In the present study, all diet arms were designed to reduce food additive intake, and we estimated the change in food additive intake as a secondary measure of adherence. Food additive intake decreased in patients on the WFD and UCD but not the CDED. This may reflect the intake of oral nutrition supplements, which contain emulsifiers and other food additives, and were categorised as flavoured milk-based drinks in the food additive FFQ. Oral nutrition supplements are part of the CDED and the sole source of nutrition in EEN. Both diets, therefore, contain food additives, yet effectively induce remission in Crohn’s disease. More research is needed to understand the different effects of food additives and their consumption in the context of other food components and overall diet quality.

### 4.2. Diet Satisfaction

Evidence for satisfaction of diet therapy options in IBD is limited. In this study, the DSAT-28 was chosen to assess diet satisfaction because of its ability to simultaneously determine preoccupation with food and several factors that influence food choice (e.g., cost, planning and preparation, perceived healthfulness/benefits of a diet). Total diet satisfaction remained high on all diet arms, including for those participants who completed the DSAT-28 at 12 months. Levels of total diet satisfaction were comparable to those achieved on a ketogenic and Mediterranean diet, in which the DSAT-28 was also used [64].

“Preoccupation with Food” did not change from week 1 to week 12 on any diet arm, with median ‘neutral’ agreement to statements in this subscale. This sub-section was of particular interest considering the ongoing caution that prescriptive diets may increase the risk of avoidant/restrictive food intake disorder and orthorexia, conditions that are known to be common in patients with IBD [13]. These findings may be influenced by our decision to exclude participants with diagnosed eating disorders. In clinical practice, patients with comorbid eating disorders and IBD should be managed by credentialed eating disorder dietitians who prioritise evidence-based frameworks that support eating disorder recovery.

Interestingly, satisfaction scores for “Eating Out” significantly increased on the CDED, which is likely explained by the fact that this diet becomes more liberal over time. Likewise, satisfaction scores for “Planning and Preparation” and “Healthy Lifestyle” significantly increased on the CDED wholefood diet, which may reflect that patients gained new ideas regarding foods and experienced symptom reduction, translating to more positive feelings regarding their new diet.

Dietary adherence is pivotal to the success of dietary therapy [5]. Likewise, there is increased awareness amongst health professionals about the importance of acknowledging IBD patients’ perspectives and experiences, with data indicating that patients have a desire for more information and active participation in their IBD care [65]. Taken together, our adherence and satisfaction data indicate that offering a range of diet strategies, using a shared decision-making model, could improve IBD patients’ experiences of routine clinical care.

### 4.3. Clinical Efficacy

In the present program, clinical efficacy was assessed based on symptom-based disease scores (CDAI, partial Mayo) and biochemical markers of inflammation (CRP, faecal calprotectin).

In patients following the CDED, there was a significant decline in disease activity from baseline to week 12 as measured by the CDAI, a statistically significant reduction in CRP, and a numerical but not statistically significant reduction in calprotectin over this time. Our findings in relation to calprotectin, which differ from previously published reports that demonstrate calprotectin response [25], may relate to the median baseline calprotectin, which was elevated (>50) but lower than in other studies.

In patients following the WFD, there was a statistically significant, but possibly not clinically significant, reduction in symptom-based disease activity indices (CDAI, partial Mayo). There was no significant decline in biochemical markers. The WFD was designed by the DELECTABLE team for this program and has similarities to the Mediterranean diet and other wholefood diets, which have been reported to reduce biochemical markers in (although necessarily induce biochemical remission) in active IBD [29,34,62,66]. The lack of significant reduction in biochemical markers in our program may be related to the small sample size and the fact that over half the patients enrolled in the WFD did not have elevated faecal calprotectin or CRP at enrolment, which could lead to a lack of demonstrable change for the whole cohort. Of note, our program focused on providing diet therapy to interested patients who do not typically meet inclusion criteria for controlled studies, and biochemical remission was not a primary outcome. Diet therapies that improve objective markers of inflammation without inducing remission may warrant consideration as adjunctive and maintenance therapies.

IBD has been associated with a reduced quality of life (QoL), including in patients with inactive disease [67], for whom residual symptoms and long-term medication use can have negative impacts. In the present study, total QoL scores (measured using the IBDQ-9) improved across all diet arms, with the largest benefit related to not having to cancel plans due to bowel problems. Increases in IBDQ-9 scores were also reported in an autoimmune protocol diet in patients with IBD [68], and a high-fibre, low-fat diet in patients with ulcerative colitis [69]. Likewise, increases in food-related quality of life have been reported in Crohn’s disease patients following an emulsifier-restricted diet [70]. Improvement in QoL scores likely relates to symptom reduction, additional supportive care, and patients’ belief that they are being proactive in improving their health.

### 4.4. Strengths

The key strength of this study is that it reflects clinical practice, enrolling patients who were keen to consider dietary therapy, irrespective of disease activity, with a focus on shared decision-making. Further, we formally assessed IBD patients’ interest in pursuing long-term, structured dietary care. Validated patient-reported tools and objective outcome measures were used. The strengths of the data lie in being real-world, prospective, and protocol-driven, with multiple time points for observation.

### 4.5. Limitations

Limitations of this study include heterogeneity in the recruited population, including patients with variable disease activity. Concurrent medical therapy was also allowed, including changes to medications. However, this was purposeful, as we aimed to assess the feasibility of running a dietary program in an IBD clinic. The UCD had low enrolment rates, with patients favouring the less restrictive WFD. Many Crohn’s disease patients were in clinical remission at study entry, which may reflect clinician referral bias, acknowledging that access to clinical trials and medications may impact recruitment to dietary therapy. This program was undertaken predominantly during the COVID-19 pandemic, which impacted the collection of objective disease markers, such as faecal calprotectin.

## 5. Conclusions

In summary, the implementation of well-balanced, effective diet therapies is well-accepted by IBD patients. Diet adherence and satisfaction are high after 3 months of dietary intervention, with a promising impact on QoL and positive disease activity trends. Positive patient experience and outcomes are achievable with moderate nutritional restrictions and should be considered as part of routine clinical care in IBD. However, to better inform future clinical practice, comparative studies and studies that elucidate patient factors (e.g., preference, habitual diet) that are key to successful disease control and long-term adherence are required.

## Figures and Tables

**Table 1:** Delectable program patient demographics at baseline.

	Whole Cohort (*n* = 64)	CDED (*n* = 15)	WF Diet (*n* = 42)	UC Diet (*n* = 7)	*p* Value
Age, Mean ± SD	36.2 ± 12.7	39.4 ± 12.8	35.5 ± 12.9	34.0 ± 3.8	0.549
Sex *Male*, *n* (%) *Female*, *n* (%)	34 (53.1%) 30 (46.9%)	10 (66.7%) 5 (33.3%)	21 (50.0%) 21 (50.0%)	3 (42.9%) 4 (57.1%)	0.457
Country of birth, *n* (%) *Australia* *Outside Australia*	54 (84.4%) 10 (15.6)	13 (86.7%) 2 (13.3%)	35 (78.6%) 7 (16.7%)	6 (85.7%) 1 (14.3%)	0.949
BMI (kg/m^2^) Median (IQR)	24.2 (5.7)	24.3 (6.2)	25.2 (4.9)	22.9 (3.9)	0.484
Disease type, *n* (%) *Crohn’s disease* *Ulcerative colitis* *Pouchitis* *Microscopic colitis*	37 (57.8%) 25 (39.1%) - 2 (3.1%)	15 (100%) ^+^ - - -	22 (52.4%) 18 (42.9%) - 2 (4.8%)	- 7 (100%) -	**<0.001** ^+^
Age at diagnosis *Median* (*IQR*)	27.0 (7.0)	34.0 (19.0)	24.0 (12.0)	30.5(12.0)	0.070
Years since diagnosis Median (IQR)	4.0 (7.0)	3.0 (5.0)	4.0 (10.0)	3.0 (5.0)	0.327
IBD drug therapy, *n* (%)					
*No treatment*	14 (21.9%)	5 (33.3%)	9 (21.4%)	0 (0%)	
*Single therapy*	31 (48.4%)	6 (40.0%)	23 (54.8%)	2 (28.6%)	**0.027 ^€^**
*Combination therapy*	5 (29.7%)	4 (26.7%)	10(23.8%)	5 (71.4%)	
Therapy type, *n* (%) *Topical treatment* *Systemic ASA* *Systemic steroid* *Immunosuppressant* *Biologic*	11 (15.1%) 18 (24.7%) 6 (8.2%) 13 (17.8%) 25(34.2%)	2(15.4%) 2 (15.4%) 2 (15.4%) 2 (15.4%) 5 (38.5%)	6 (13.0%) 12 (27.9%) 3(7.0%) 10 (23.3%) 15 (34.9%)	3(21.4%) 4(28.6%) 1 (7.1%) 1 (7.1%) 5 (35.7%)	0.616

BMI = Body Mass Index; ^€^ Assessed for highest level of drugtherapy; ^+^ CDED and UC diet differ across disease types based on an assessment of adjusted residuals. ^€^ UC differs from WF (*p* = 0.013) and CDED (*p* = 0.010).

**Table 2:** Additive intake (mg/day) at baseline and week 12 on the Crohn’s Disease Exclusion Diet, the Wholefood Diet, and the Ulcerative Colitis Diet in the DELECTABLE program.

	Timepoint	CDED (*n* = 9) Median [IQR]	*p* Value	WF Diet (*n* = 26) Median [IQR]	*p* Value	UC Diet (*n* = 7) Median [IQR]	*p* Value
Total Additive intake	Baseline	4032.4 [7239.6]	0.441	3229.3 [4847.3]	0.009	12,625.9 [1811.6]	0.018
	Week 12	2491.1 [6619.8]		1580.7 [3647.2]		2796.5 [4618.6]	
Aspartame	Baseline	245.6 [737.9]	0.374	264.3 [315.9]	0.096	866.3 [2522.1]	0.043
	Week 12	222.9 [344.7]		128.5 [310.8]		142.7 [282.4]	
Carboxymethylcellulose	Baseline	1117.0 [500.0]	0.484	1279.1 [1759.8]	0.002	2853.3 [2534.2]	0.018
	Week 12	157.8 [1934.6]		162.6 [551.4]		394.3 [2221.0]	
Carrageenan	Baseline	1272.8 [2066.8]	0.441	1052.8 [1285.4]	0.013	2153.3 [4484.6]	0.028
	Week 12	766.1 [1983.0]		387.1 [1486.4]		737.5 [763.3]	
Polysorbate-80	Baseline	747.4 [1842.1]	0.374	466.9 [763.5]	0.041	2426.7 [2752.0]	0.028
	Week 12	556.6 [898.6]		299.6 [754.6]		307.4 [827.9]	
Saccharin	Baseline	123.0 [238.0]	0.374	85.5 [80.8]	0.052	232.2 [698.2]	0.018
	Week 12	80.3 [200.8]		55.6 [99.4]		97.6 [193.3]	
Sucralose	Baseline	110.0 [368.4]	0.374	136.5 [167.9]	0.018	743.7 [2029.5]	0.028
	Week 12	142.6 [192.2]		74.6 [139.3]		69.9 [651.9]	
Sulphites	Baseline	40.2 [116.4]	0.767	36.4 [150.8]	0.007	120.6 [421.4]	0.018
	Week 12	39.6 [54.6]		14.7 [33.7]		15.2 [74.7]	
Titanium dioxide	Baseline	114.1 [212.2]	0.889	32.7 [68.9]	0.443	149.5 [430.1]	0.018
	Week 12	85.4 [174.2]		44.0 [107.7]		33.0 [52.6]	

**Table 3:** Disease activity scores and biochemical indices at baseline, week 6, and week 12 on the DELECTABLE program.

	CDED				WFD				UCD		
Variable	Baseline	Week 6	Week 12	*p* Value	Baseline	Week 6	Week 12	*p* Value	Baseline	Week 6	Week 12
CRP (mg/L), Median [IQR]	7.5 [15.5]	5.0 [6.0]	4.6 [7.3] c	0.043	3.6 [5.6]	2.0 [5.0]	1.9 [5.8]	0.545	0.0 [2.5]	1.0 [2.0]	1.0 [2.7]
CRP (mg/L), *n* (%)				a, 1.00				a, 0.69			
<5 (normal)	6 (40%)	4 (40%)	6 (46.2%)	b, 1.00	6 (85.7%)	6 (85.7%)	5 (83.3%)	b, 1.00	6 (85.7%)	6 (85.7%)	5 (83.3%)
≥5 (elevated)	9 (60%)	6 (60%)	7 (53.8%)	c, 1.00	1 (14.3%)	1 (14.3%)	1 (16.7%)	c, 0.344	1 (14.3%)	1 (14.3%)	1 (16.7%)
Calprotectin, Median [IQR]	195.0 [560.0]	123.0 [98.0] a	62.0 [228.0] c	0.062	17.6 [237.4]	11.3 [237.4]	17.8 [88.2]	0.958	112.0 [−]	46.0 [−]	174.0 [−]
Calprotectin, *n* (%)				a, n/a				a, 1.00			
<50 (normal)	1 (6.7%)	1 (11.1%)	5 (45.5%)	b, 1.000	22 (52.4%)	14 (58.3%)	16 (61.5%)	b, 0.69	3 (42.9%)	2 (66.7%)	5 (71.4%)
≥50 (elevated)	12 (92.3%)	8 (58.9%)	6 (54.5%)	c, 1.000	7 (40.5%)	10 (41.7%)	10 (38.5%)	c, 1.00	4 (57.1%)	1 (33.3%)	2 (28.6%)
CDAI, Median [IQR]	153.0 [172.0]	61.8 [103.0] a	43.1 [84.0] c	0.023	49.5 [92.0]	38.0 [40.0] a	31.8 [77.0] c	0.038	-	-	-
CDAI, *n* (%)				a, 0.031				a, 0.037	n/a	n/a	n/a
Remission (0–149)	8 (53.3%)	12 (92.3%)	13 (86.7%)	b, 1.00	20 (87%)	15 (93.8%)	20(90.9%)	b, 1.00	n/a	n/a	n/a
Active disease (>149)	7 (46.7%)	1 (7.7%)	2 (13.3%)	c, 0.063	3 (13%)	1 (6.3%)	2 (4.8%)	c, 1.00	n/a	n/a	n/a
Partial Mayo, Median [IQR]	n/a	n/a	n/a	n/a	2.0 [4.0]	0.0 [4.0] a	0.0 [1.0] c	0.02	3.0 [5.0]	3.0 [3.0]	3.0 [3.0]
Partial Mayo, *n* (%)	n/a	n/a	n/a	n/a				a, >0.001			
Remission (0–1)	n/a	n/a	n/a	n/a	5 (26.3%)	15 (72.2%)	15 (78.9%)	b, 0.625	3 (42.9%)	3 (42.9%)	3 (42.9%)
Active disease (>1)	n/a	n/a	n/a	n/a	14 (73.7%)	5 (27.8%)	4 (21.1%)	c, 0.002	4 (57.1%)	5 (57.1%)	6 (57.1%)

CRP = C-reactive protein. CDAI = Crohn’s Disease Activity Index. a = Difference between baseline and week 6; b = Difference between week 6 and week 12; c = Difference between baseline and week 12. n/a = Not applicable.

## Data Availability

The participants of this study did not give written consent for their data to be shared publicly, so, due to the sensitive nature of the research, supporting data are not available.

## Supplementary Materials

The following supporting information can be downloaded at: https://www.mdpi.com/article/10.3390/nu17091592/s1, Table S1: Factors influencing diet selection in the DELECTABLE program; Table S2: Dietary analysis of DELECTABLE program diets; Table S3: Dietitian- and patient-rated adherence; Table S4: Additional food frequency questionnaire items; Table S5: Modified DSAT-28 Items; Table S6: Dietitian- and patient-rated adherence at baseline, week 6, and week 12 on the CDED, the Wholefood Diet, and the Ulcerative Colitis Diet; Table S7: Diet satisfaction (Modified DSAT-28) and quality of life (IBDQ-9) at week 1 and week 12 on the CDED, the Wholefood Diet, and the Ulcerative Colitis Diet; Table S8: Change in CRP, calprotectin, CDAI, and partial Mayo for patients with elevated baseline values; Table S9: Medication changes from baseline to week 12.

## Abbreviations

The following abbreviations are used in this manuscript:

CDAI	Crohn’s Disease Activity Index
CDED	Crohn’s Disease Exclusion Diet
CRP	C-reactive Protein
EEN	Exclusive Enteral Nutrition
IBD	Inflammatory Bowel Disease
RCT	Randomised Control Trial
UCD	Ulcerative Colitis Diet
WFD	Wholefood Diet
