# Supplementary material for: Practical Application of Evidence-Based Dietary Therapy in Inflammatory Bowel Disease: The DELECTABLE Program
Source: Nutrients. 2025 May 6;17(9):1592. doi: 10.3390/nu17091592 (PMC12073524; doi:10.3390/nu17091592)
Supplement: Supplementary file 1 [file nutrients-17-01592-s001.zip › nutrients-3581836-supplementary.pdf]

SUPPLEMENTARY TABLES

Supplementary Table S1: Factors influencing diet selection in the DELECTABLE program

Crohn's Disease Exclusion Diet	***Whole Food Diet***	***Ulcerative Colitis Diet***
Crohn's Disease or Pouchitis	Crohn's Disease, Ulcerative colitis, Microscopic Colitis, Pouchitis (*ANY CONDITION)*	Ulcerative colitis or Microscopic Colitis
Patient prefers a strict approach	Patient prefers a moderate approach	Patient prefers a strict approach
Patient is willing to include chicken and eggs in their diet	Patient is vegetarian or vegan	Patient is vegetarian or vegan
Patient has a stricture and or/severe disease	Patient has moderate or inactive disease	Patient has moderate or inactive disease
Patient is newly diagnosed and/or would like to avoid/reduce medication	Patient has recently trialled EEN or CDED and would like to transition to a more liberal diet long-term or Patient cannot tolerate oral nutrition supplements	

Supplementary Table S2: Dietary analysis of DELECTABLE Program diets

	**Wholefood Diet**	**Ulcerative Colitis Diet**	**Crohn's Disease Exclusion Diet**
**Energy**	8419 – 10 977 kJ	86468 – 11 200 kJ	9200 – 11 200 kJ
**Energy contribution from macronutrients**<br><br>*Recommended -*<br>*15-25% protein*<br>*25-35% fat*<br>*45-65% carbohydrate*	20-21% Protein (2g/kg)<br>26-27% Fat (8% saturated fat)<br>46-47% Carbohydrate	16% Protein (1.6g/kg)<br>21% Fat (5% saturated fat)<br>57% Carbohydrate	19-20% Protein (1.9g/kg)<br>30% Fat (5% saturated fat)<br>46-47% Carbohydrate
**Fibre**<br><br>*Recommended 25-30g*<br>*No Upper Limit*	36.2 - 44.6g	44g	20.9g -24.1g
**Unsaturated: saturated fatty acid ratio**<br>*Recommended >2:1*<br><br>**Omega 6: Omega 3 ratio**<br>*Recommended < 4:1; Western diet 10:1 to 50:1*	~2:1<br>(32%SFA, 45-48% MUFAS, 20-24%PUFA)<br><br>~3:1	~2:5<br>(28%SFA, 48%MUFAS, 24%PUFAS)<br><br>~3:1	~2:1<br>(17%SFA; 58%MUFAS; 25%PUFA)<br><br>~4: 1 to 5:1
**Micronutrient adequacy**	Exceeds RDI and AI for all nutrients for males; Exceeds RDI and AI for all nutrients except iron for females (still meats EAR for iron but supplement will be recommended for some)	Exceeds RDI and AI for all nutrients for males; Exceeds RDI and AI for all nutrients except iron for females (still meats EAR for iron but supplement will be recommended for some)	Exceeds RDI for all nutrients expect calcium in females (calcium supplement recommended in CDED paper)<br>Exceeds AI for all nutrients except fibre, which is intentional
**Food groups**<br>*Recommended:*<br>*Min 6 grains*<br>*Min 2 fruits*<br>*Min 5-6 veg*<br>*Min 3 dairy*<br>*Min 2.5 – 3 protein*	7.2 to 9.8 serves grains<br>2.4 to 2.5 serves fruits<br>5.8 to 6.1 serves vegetables<br>3.2 serves dairy<br>2.6 to 3.8 serves proteins<br>*(1.4 to 2.0 animal)*	7.11 serves grains<br>3.4 serves fruits<br>6.2 serves vegetables<br>2.1 serves dairy<br>1.6 serves proteins<br>(*0.7 animal protein)*	1.76 -2.54 serves grains<br>2.94 serves fruits<br>5.8 serves vegetables<br>0 serves dairy<br>2.4 serves proteins<br>(*all animal)*

Supplementary Table S3: Dietitian and Patient-rated adherence

**Dietitians assessment of adherence**<br>Is the patient adherent with dietary therapy?					
☐ Very adherent<br>☐ Fairly adherent<br>☐ Partially adherent<br>☐ Non-adherent but will try to adhere to diet<br>☐ Non-adherent will not adhere to diet					
**Participants assessment of adherence**<br>Please indicate if you have followed the diet instructions in the way you have been asked to since your last appointment					
☐Never	☐ Rarely	☐ Sometimes	☐ Often	☐ Very often	☐ Always
**Since the beginning of the study:**					
I forgot to follow the diet instructions				☐ Yes	☐ No
I stopped following the diet instructions for a while				☐ Yes	☐ No
I only followed the diet instructions when I had active symptoms				☐ Yes	☐ No
I decided to ignore the diet for a meal				☐ Yes	☐ No
I took less formula than instructed				☐ Yes	☐ No
I took more formula than instructed				☐ Yes	☐ No
I avoided following the diet instructions if I could				☐ Yes	☐ No
I followed the diet instructions regularly everyday				☐ Yes	☐ No

Supplementary Table S4: Additional Food Frequency Questionnaire Items

A1. How often do you choose organic fruits and vegetables?	Always/Mostly/Sometimes/Never
A2. How often do you choose gluten free products (this refers to processed foods only, not naturally gluten-free foods)?	Always/Mostly/Sometimes/Never
A3. How often do you choose low fat products (this refers to processed foods only, not naturally low-fat foods)?	Always/Mostly/Sometimes/Never
A4. How often do you eat foods from local restaurants (e.g., Asian, Italian, Mexican)?	Daily/Weekly/Most months/Every few months/Once to twice per year/Never
A5. How often do you eat Fast Foods (E.g. McDonalds, KFC, Pizza Hut, Lord of the Fries)?	Daily/Weekly/Most months/Every few months/Once to twice per year/Never

Table note: The food frequency questionnaire utilised in this program has previously been published [2]

Supplementary Table S5: Modified DSAT-28 Items

**Compared to your diet before starting this special IBD diet…**	
**Healthy Lifestyle Factor (n =5)**	
I believe that I am reducing my risk for disease by the way that I eat.	Strongly agree/ agree/ neutral/ disagree/ strongly agree
I feel good about myself.	Strongly agree/ agree/ neutral/ disagree/ strongly agree
I think that I eat a healthy diet.	Strongly agree/ agree/ neutral/ disagree/ strongly agree
I think that I have a healthy lifestyle.	Strongly agree/ agree/ neutral/ disagree/ strongly agree
I am satisfied with my current diet.	Strongly agree/ agree/ neutral/ disagree/ strongly agree
**Eating out Factor (n =4)**	
*Finding appropriate food choices at restaurants is difficult.	Strongly agree/ agree/ neutral/ disagree/ strongly agree
The way I currently eat prevents me from eating in restaurants frequently.	Strongly agree/ agree/ neutral/ disagree/ strongly agree
*I have difficulty finding the foods I want when eating out.	Strongly agree/ agree/ neutral/ disagree/ strongly agree
When dining out, I can easily choose foods from the menu that fit into my current diet.	Strongly agree/ agree/ neutral/ disagree/ strongly agree
**Cost Factor (n =)**	
*I feel that I spend a large amount of my budget on the foods that I eat.	Strongly agree/ agree/ neutral/ disagree/ strongly agree
*I think that preparing food and meals for the way I eat now costs a lot of money.	Strongly agree/ agree/ neutral/ disagree/ strongly agree
*I spend a lot of money on food.	Strongly agree/ agree/ neutral/ disagree/ strongly agree
*It is hard for me to afford the kind of foods that I eat.	Strongly agree/ agree/ neutral/ disagree/ strongly agree
I think that preparing food and meals for the way I eat now is economical.	Strongly agree/ agree/ neutral/ disagree/ strongly agree
**Planning and Preparation Factor (n = 5)**	
*I think preparing food and meals for the way I eat now requires a lot of effort.	Strongly agree/ agree/ neutral/ disagree/ strongly agree
*I spend a lot of time shopping for food.	Strongly agree/ agree/ neutral/ disagree/ strongly agree
*I spend a lot of time planning my meals.	Strongly agree/ agree/ neutral/ disagree/ strongly agree
*I think preparing food and meals for the way I eat now is time-consuming.	Strongly agree/ agree/ neutral/ disagree/ strongly agree
*I spend a lot of time looking for new ideas for food and meals that fit into my current diet.	Strongly agree/ agree/ neutral/ disagree/ strongly agree
**Pre-occupation with Food Factor (n =6)**	
*I feel that my diet controls my life.	Strongly agree/ agree/ neutral/ disagree/ strongly agree

*I have cravings for some of my favourite foods that are not allowed on my current diet.	Strongly agree/ agree/ neutral/ disagree/ strongly agree
*I think about food between almost every meal.	Strongly agree/ agree/ neutral/ disagree/ strongly agree
*I often feel hungry.	Strongly agree/ agree/ neutral/ disagree/ strongly agree
*Thoughts of food are always on my mind.	Strongly agree/ agree/ neutral/ disagree/ strongly agree
*I always feel like I want to snack between meals.	Strongly agree/ agree/ neutral/ disagree/ strongly agree
**IBD-specific Factor (n =3)**	
*The flavour of meals and snacks on my new diet is less appetising	Strongly agree/ agree/ neutral/ disagree/ strongly agree
I will be able to maintain my current diet in the long term	Strongly agree/ agree/ neutral/ disagree/ strongly agree
The way I currently eat has reduced gastrointestinal symptoms (e.g. bloating and gas, pain)	Strongly agree/ agree/ neutral/ disagree/ strongly agree
*Reverse scored item	

Supplementary Table S6: Dietitian and Patient rated adherence at Week Baseline, Week 6 and Week 12 on the CDED, Wholefood Diet and Ulcerative Colitis Diet

	**Crohn's Disease Exclusion Diet**				**Wholefood Diet**				***Ulcerative Colitis Diet***		
Variable (possible range), Median [IQR]	*Week 1*	*Week 6*	*Week 12*	*P*	*Week 1*	*Week 6*	*Week 12*	*P*	*Week 1*	*Week 6*	*Week 12*
**Dietitian-Rated Adherence (0-4)**	4 [2]	3 [2]	3 [1]	*0.565*	3 [1]	3 [1]	3 [1]	*0.165*	4 [1]	3.5 [2]	4 [1]
Non-compliant will not adhere	0, 0%	0, 0%	0, 0%		0, 0%	0, 0%	0, 0%		0, 0%	0, 0%	0, 0%
Non-compliant will try to adhere	0, 0%	1, 7.7%	0, 0%		2, 4.8%	1, 2.7%	0, 0%		0, 0%	0, 0%	0, 0%
Partially compliant	3, 20%	2, 5.4%	2, 5.4%		7, 17.5%	6, 16.2%	8, 20%		1, 4.3%	2, 3.3%	0, 0%
Fairly complaint	2, 6.7%	5, 8.5%	5, 8.5%		18, 45 %	13, 5.1%	16, 40%		2, 8.6%	1, 6.7%	2, .6%
Very complaint	8, 3.3%	5, 8.5%	6, 6.2%		13, 2.5%	4, 45.9%	4, 40%		3, 7.1%	3, 0.0%	5, .4%
**Patient-Rated Adherence (0-5)**	5 [1]	4 [2]	4 [1]	*0.682*	4 [3]	4 [3]	4 [2]	*0.348*	4.5 [1]	4.5 [1]	4 [0]
Never	0, 0%	0, 0%	0, 0%		0, 0%	0, 0%	0, 0%		0, 0%	0, 0%	0, 0%
Rarely	0, 0%	0, 0%	0, 0%		2, 5%	1, 2.7%	0, 0%		0, 0%	0, 0%	0, 0%
Sometimes	1, 7.1%	3, 1.4%	1, 7.7%		5, 12.5%	6, 16.2%	5, 12.8%		0, 0%	0, 0%	0, 0%
Often	2, 4.3%	1, 7.1%	1, 7.7%		6, 15%	7, 18.9%	8, 20.5%		1, 4.3%	1, 6.7%	0, 0%
Very Often	5, 5.7%	4, 8.6%	7, 3.8%		18, 45%	11, 9.7%	16, 41%		3, 2.9%	2, 3.3%	7, 100%
Always	6, 40%	6, 2.9%	4, 0.8%		9, 22.5%	11, 9.7%	10, 5.8%		3, 2.9%	3, 50%	0, 0%

Supplementary Table S7: Diet Satisfaction (Modified DSAT-28) and Quality of Life (IBDQ-9) at Week 1 and Week 12 on the CDED, Wholefood Diet and Ulcerative Colitis Diet

Variable (possible range), Mean +/- SD	**Crohn's Disease Exclusion Diet**			**Wholefood Diet**			***Ulcerative Colitis Diet***	
	Week 1	*Week 12*	*P*	*Week 1*	*Week 1*	*P*	*Week 1*	*Week 12*
Modified DSAT-28 Total Score *(27-135)*	79.0 ±13.3	84.1±13.3	*0.212*	84.1±12.2	86.9±11.7	***0.033***	88.4±5.4	88.4±5.5
Total mean score per question	2.9	3.1		3.1	3.2		3.3	3.3
Healthy Lifestyle Category Score (5-25)	19.9 ±2.6	20.1±2.2	*0.168*	19.7±2.6	20.6±2.7	***0.050***	20.0±1.9	20.0±2.7
Healthy Lifestyle mean score per question	4.0	4.0		3.9	4.1		4.0	4.0
Eating Out Category Score (4 -20)	6.4±2.4	9.7±3.0	***0.005***	10.4±3.1	11.3±3.8	*0.207*	9.0±3.8	9.2±3.0
Eating out mean score per question	1.6	2.4		2.6	2.8		2.3	2.3
Cost Category Score (5-25)	16.3±3.4	16.3±4.2	*0.669*	16.4±3.1	16.6±3.4	*0.459*	18.6±4.3	18.0±27
Cost mean score per question	3.3	3.3		3.3	3.3		3.7	3.6
Food preoccupation mean category score (6 -30)	17.0±4.0	19.0±3.2	*0.409*	18.8±3.9	19.1±3.9	*0.415*	18.6±3.5	20.2±4.7
Food preoccupation mean score per question	2.8	3.2		3.1	3.2		3.1	3.4
Planning & preparation category score (4-20)	12.2±11.0	11.2±2.4	*0.531*	11.1±3.1	12.1±2.8	***0.008***	13.2±4.7	13.0±3.1
Planning and Preparation mean score per questionnaire	3.1	2.8		2.8	3.0		3.3	3.3

IBD mean question score (3-15)	9.7±2.5	11.0±1.9	*0.062*	11.0±2.1	11.2±2.0	*0.168*	12.0±0.7	12.0±1.0
IBD mean score per question	3.2	3.7		3.7	3.7		4.0	4.0
*IBDQ9 (7-49) total score	35.4 ±10.0	50.8 ±7.1	***<0.001***	37.1±9.6	48.3±9.6	***<0.001***	39.6±5.9	41.0±6.0
IBDQ-9 mean score per question	3.9	5.6		4.1	5.4		4.4	4.6

Supplementary Table S8: Change in CRP, Calprotectin, CDAI and Partial Mayo for patients with elevated baseline values

	Crohn's Disease Exclusion Diet				Wholefood Diet			
Variable (possible range), Median [IQR]	*Week 1*	*Week 6*	*Week 12*	*P*	*Week 1*	*Week 6*	*Week 12*	*P*
**CRP**	14 [15.0]	5 [11.0]	5 [11.0][c]	*0.022*	6 [7.8]	5 [4.0]	6 [8.3]	*0.232*
**Calprotectin**	195 [560.0] [a]	123 [98]	62 [228][c]	*0.062*	323 [395.7]	90.8 [449.7]	54.6 [127.5][c]	*0.072*
**CDAI (CDED: n =7; WFD: n =3)**	222.0 [68]	104.0 [73]	77.0 [135]		273.0 [-]	102.0 [-]	105 [111]	-
**Partial Mayo**					3 [3] [a]	1[4]	3[3] [c]	***<0.01***

Supplementary Table S9: Medication changes from baseline to week 12 [3, 4]

	Whole cohort	Active disease at enrolment (n=44)		Remission at enrolment (n=20)	
		CD	UC	CD	UC
**Change in medication**	26	7	11	4	4
**Commenced medication**	9	6<br>*Steroids n=2*<br>*Biologic n=4*	2<br>*Topical n=1*<br>*Biologic n=1*	-	2<br>*Steroids n=1*
**Dose Reduction**	3	3	2	1	-
**Ceased medications**	15	3<br>*Steroids n=1, immunosuppression n=2*	6<br>*Steroids n=6*	3<br>Steroids n=1<br>6MP n=2	2<br>Steroids n=2

REFERENCES

1. Levine A, Rhodes JM, Lindsay JO, et al. Dietary guidance from the international organization for the study of inflammatory bowel diseases. *Clin Gastroenterol Hepatol*. 2020;18: 1381-92.
2. Trakman GL, Lin WYY, Hamilton AL, et al. Processed Food as a Risk Factor for the Development and Perpetuation of Crohn's Disease-The ENIGMA Study. *Nutrients*. 2022;14.
3. Day AS, Yao CK, Costello SP, et al. Food avoidance, restrictive eating behaviour and association with quality of life in adults with inflammatory bowel disease: A systematic scoping review. *Appetite*. 2021;167: 105650.
4. Holt DQ, Strauss BJ, Moore GT. Patients with inflammatory bowel disease and their treating clinicians have different views regarding diet. *J Hum Nutr Diet*. 2017;30: 66-72.
